# Optimization of the Clinical Effectiveness of Radioembolization in Hepatocellular Carcinoma with Dosimetry and Patient-Selection Criteria

**DOI:** 10.3390/curroncol29040196

**Published:** 2022-03-29

**Authors:** Philippe d’Abadie, Stephan Walrand, Renaud Lhommel, Michel Hesse, Ivan Borbath, François Jamar

**Affiliations:** 1 Department of Nuclear Medicine, Cliniques Universitaires Saint Luc, Université Catholique de Louvain, 1200 Brussels, Belgium; stephan.walrand@uclouvain.be (S.W.); renaud.lhommel@uclouvain.be (R.L.); michel.hesse@uclouvain.be (M.H.); francois.jamar@saintlluc.uclouvain.be (F.J.); 2 Department of Gastroenterology, Cliniques Universitaires Saint Luc, Université Catholique de Louvain, 1200 Brussels, Belgium; ivan.borbath@uclouvain.be

**Keywords:** liver radioembolization, selective internal radiation therapy, hepatocellular carcinoma, dosimetry, optimization

## Abstract

Selective internal radiation therapy (SIRT) is part of the treatment strategy for hepatocellular carcinoma (HCC). Strong clinical data demonstrated the effectiveness of this therapy in HCC with a significant improvement in patient outcomes. Recent studies demonstrated a strong correlation between the tumor response and the patient outcome when the tumor-absorbed dose was assessed by nuclear medicine imaging. Dosimetry plays a key role in predicting the clinical response and can be optimized using a personalized method of activity planning (multi-compartmental dosimetry). This paper reviews the main clinical results of SIRT in HCC and emphasizes the central role of dosimetry for improving it effectiveness. Moreover, some patient and tumor characteristics predict a worse outcome, and toxicity related to SIRT treatment of advanced HCC patient selection based on the performance status, liver function, tumor characteristics, and tumor targeting using technetium-99m macro-aggregated albumin scintigraphy can significantly improve the clinical performance of SIRT.

## 1. Introduction

Liver radioembolization (RE) or selective internal radiation therapy (SIRT) is part of the treatment strategy for hepatocellular carcinoma (HCC) [1]. This treatment involves the injection of radioactive microspheres via the liver arterial blood supply of the tumor(s). These microspheres are trapped in the arterioles of the tumor(s) and the targeted liver parenchyma. The liver parenchyma is primarily supplied by the portal vein, while HCC perfusion is primarily supplied by the hepatic arteries. This preferential vascularization allows a high irradiation of tumors while limiting radiation of the healthy liver [2]. The tumor-absorbed dose can range from 100 to 1000 Gy [3]. In comparison, the dose that can be delivered to tumors is limited to a maximum of 70 Gy, with external beam radiotherapy to avoid irreversible liver damage [4]. Yttrium-90 (^90^Y)-resin microspheres (Sir-Spheres^®^; Sirtex Medical Ltd., Sydney, Australia), ^90^Y-glass microspheres (Therasphere^®^; Boston Scientific, Boston, MA, USA), and holmium-166-poly-L-lactic acid microspheres (QuiremSpheres^®^; Quirem Medical B.V., Deventer, The Netherlands) are the three commercially available radioactive microspheres, differing by their physical and irradiation properties [5].

SIRT is planned in two phases. First, a simulation is always performed to evaluate the feasibility of the treatment. An interventional radiologist catheterizes the liver artery(ies) and evaluates the arterial feeding of the tumor(s). A non-therapeutic nuclear medicine agent, technetium-99m macro-aggregated albumin (MAA), is injected into the liver artery(ies) supplying the tumor(s) for simulating the distribution of the radioactive microspheres. Thereafter, the MAA distribution is assessed by nuclear imaging using single-photon emission computed tomography combined with computed tomography (MAA SPECT/CT). This imaging confirms the accurate targeting of the tumor(s) and the absence of risk of toxicity (digestive or lung irradiation). Then, the phase of treatment is scheduled with injection of the radioactive microspheres in the same technical conditions. The recommended methods for calculating the amount of radioactive microspheres needed for the treatment (activity) differ between the different available microspheres [6,7,8]. These methods are semi-empirical, based on the body surface area for resin microspheres, and using a mono-compartmental model (based on the liver volume) for glass and holmium-166-poly-L-lactic acid microspheres [9]. During the workup, the MAA distribution in the tumor, the healthy liver, and the lung compartments can also be evaluated to perform a more personalized method of activity planning (multi-compartmental or partition model) [1].

After therapy, the tumor and the healthy liver absorbed doses are determined with nuclear medicine imaging. With ^90^Y microspheres, the absorbed doses are ideally evaluated using positron emission tomography combined with computed tomography (^90^Y PET/CT). ^90^Y PET/CT accurately predicts the absorbed doses [10].

## 2. Clinical Results of SIRT in HCC

The treatment options for HCC depend on the Barcelona Clinic Liver Cancer (BCLC) staging system [11]. This classification takes into account the tumor characteristics (i.e., size, number of tumors, portal vein invasion, or extra hepatic spread), underlying liver function (via Child–Pugh score) and patient performance status (via Eastern Cooperative Oncology Group (ECOG) scale) [12]. The BCLC stage is a well-established accurate predictor of patient survival and in routine clinical use worldwide to help determine the best treatment options.

Recent recommendations of the European Society for Medical Oncology consider SIRT as an alternative treatment for patients with BCLC stages A, B, and C [13,14]. For BCLC stage A patients, a recent large, retrospective study demonstrated that SIRT was very efficient to address unresectable solitary HCC alone or for use as a neoadjuvant bridge in a curative surgical approach [15]. For intermediate HCC (i.e., BCLC B), transarterial chemoembolization is recommended for first-line therapy. However, a meta-analysis of previous prospective randomized studies comparing SIRT to transarterial chemoembolization demonstrated similar survival outcomes [16]. Moreover, a randomized study comparing SIRT to transarterial chemoembolization in a population of BCLC A and B patients demonstrated similar survival times but showed that the former was associated with a longer time to progression [17].

Considering advanced stage patients (i.e., BCLC C), systemic therapies are often preferred; these include immunotherapy (e.g., atezolizumab plus bevacizumab) or targeted therapy (e.g., sorafenib, regorafenib). Patients treated with atezolizumab plus bevacizumab demonstrated superior survival and progression-free survival compared to patients treated with sorafenib [18]. However, randomized controlled trials comparing SIRT to sorafenib have failed to demonstrate a superior outcome with SIRT [19,20,21]. Consequently, the place of SIRT in advanced HCC is an alternative and possibilities for therapy optimization should be investigated.

The main results of prospective and randomized studies published to date that have compared SIRT to alternative therapies in HCC patients are summarized in Table 1.

Controlled trials currently investigate the combination of SIRT plus immunotherapy in patients with intermediate and advanced stages of HCC. Preliminary results of the combination of nivolumab three weeks after SIRT demonstrated a favorable tolerability and encouraging response rates [24,25]. A randomized trial (NCT04541173) is also investigating the safety and effectiveness of SIRT followed by the combination of atezolizumab plus bevacizumab. In theory, the combination of immunotherapy after SIRT may give a synergistic clinical effect and improve tumor control and patient survival. Ionizing radiation may induce the release of tumor-associated antigens targeted by antigen presenting cells and result in a stimulation of the immune response, boosting the effects of immunotherapy [26]. SIRT must be performed before the initiation of immunotherapy, when the biological effects of ionizing radiations are effective.

## 3. Clinical Dosimetry in SIRT

Tumor dosimetry is a predictive factor of SIRT efficiency. Previous data have demonstrated a correlation between tumor-absorbed dose and radiological response [27,28,29]; indeed, a high tumor-absorbed dose is associated with a high probability of tumor control. In addition, a multitude of clinical data demonstrating strong correlation between tumor-absorbed dose, radiological response, and survival of HCC patients are currently available (overview in Table 2). Table 3 and Table 4 summarize the main studies reporting a tumor-absorbed dose threshold associated with SIRT efficiency in HCC. Studies comparing glass to resin microspheres have indicated that the tumor-absorbed dose cut-off is usually two-fold, which is explained by their different physical and radioactive properties [5,30].

## 4. Personalized Dosimetry in SIRT

**Figure 1:**
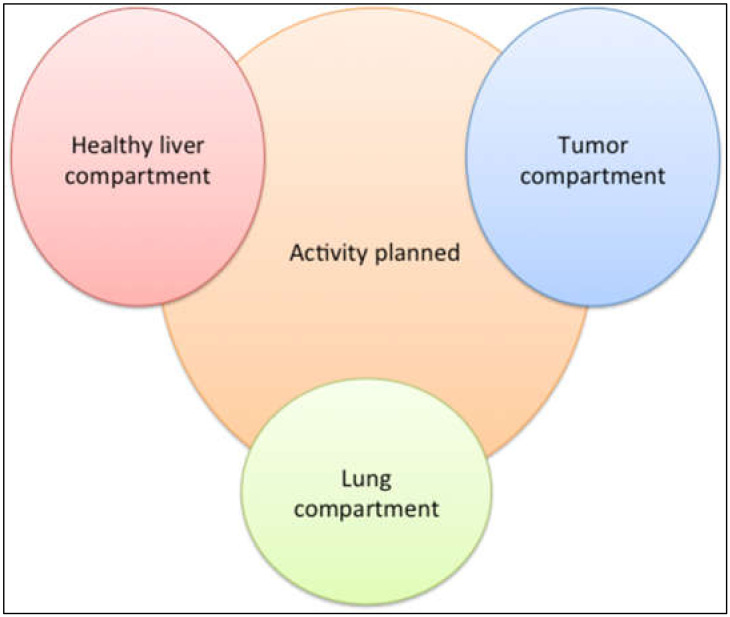
Multi-compartment dosimetry (partition model) using technetium-99m macro-aggregated albumin single-photon emission computed tomography combined with computed tomography for activity planning. The absorbed doses in these different compartments can be simulated before treatment and enable optimization of the activity planned.

To improve the clinical results of RE, the activity prescription can be more personalized and optimized to reach higher tumor-absorbed doses. As previously described, the recommended activity prescription is calculated using semi-empirical methods. While these methods are safe, they can induce suboptimal absorbed doses to tumors [41]. A recent prospective study confirmed the clinical benefits of performing multi-compartmental dosimetry (known as “the partition model”) [40]. In the partition model, activity planning is based upon the MAA distribution in the different compartments (Figure 1), simulating an absorbed dose under the threshold of toxicity for the healthy liver and above the efficacy threshold for the tumor(s).

The dose to the healthy liver can be accurately predicted with MAA SPECT/CT, controlling the risk of liver toxicity [42]. Indeed, an excess of liver radiation can induce liver damage (i.e., RE-induced liver disease). The toxicity threshold doses have been well-demonstrated through non-tumoral, whole-liver dose (reaching 90 Gy for glass microspheres and 40–50 Gy for resin microspheres) [43,44]. As such, with MAA SPECT/CT dosimetry simulating an absorbed dose to the healthy liver under these thresholds, the activity can be planned safely. Moreover, the external beam radiotherapy models have shown that no liver damage can occur if the treated liver volume does not exceed 40% [45]. When a small part of the liver volume is targeted by the treatment, the planned activity can be increased for performing a safe radiation segmentectomy. For treatments applied to a majority of the liver (>60%), the planned activity can be adjusted to reach the maximal tolerable liver absorbed dose. With this method, the planned activity would be the highest possible and would therefore increase the activity in the tumor compartment to maximize the tumor-control probability.

Moreover, a large HCC tumor size (≥5 cm) was a factor of poor prognosis in some studies [46,47,48]. These studies included patients treated by glass microspheres, using the recommended method of activity planning (80–150 Gy to the targeted liver). Given this, Garin et al. [33] demonstrated a significant lower response rate in large HCC tumors (size ≥ 5 cm) using this same method of activity planning, probably because of tumor underdosing. More interestingly, using an optimized method of activity planning increasing the tumor-absorbed dose, Garin et al. [49] demonstrated a high response rate in large HCC tumors and no correlation between the tumor size (≥5 cm) and the patient survival.

A recent prospective trial performed with patients with HCC, mostly with advanced stage disease, demonstrated better outcome achieved with personalized dosimetry and MAA imaging (using glass microspheres) [40]. When an approach reaching a maximum dose of 120 Gy to the targeted healthy liver, and at least 205 Gy to tumors (>250 Gy if possible) was used, the clinical outcome was highly improved as compared to patients treated with the standard (120 Gy to the targeted liver) dosimetric approach. The main results of this trial are summarized in Table 5. The median activity was increased by 38%, as shown upon comparison of the standard method to this personalized method of activity planning. Similarly, in a retrospective study using personalized dosimetry with a whole, normal liver dose reaching 40 to 70 Gy (glass microspheres), the median survival was 14.1 mo in HCC patients with portal vein invasion (95% confidence interval (CI): 10.7–17.5 mo) [50]. These results were higher than expected, considering other published data from a similar population treated with a standard dosimetric approach (median: 10.4 mo, 95%CI: 7.2–16.6) [48].

However, using this optimized method of activity planning, patients with risk factors for RE-induced liver disease must be carefully evaluated before treatment to limit the liver toxicity probability. For this purpose, ^99m^Tc–mebrofenin scintigraphy with SPECT/CT can evaluate and quantify the global and regional liver functions and predict the risk of post-radiation liver damage. In patients who undergo major liver resection, the remnant liver uptake of mebrofenin correlated well with the risk of postoperative liver failure (cut-off value: 2.69%/min/m^2^) [51]. This technique could also be applied to SIRT for evaluating liver function in patients with risk factors (e.g., advanced cirrhosis, large tumor involvement, etc.). Indeed, the mebrofenin liver uptake of the non-treated liver was also predictive of RE-induced liver disease in some case series [52,53].

## 5. Optimization of Tumor Targeting

Better tumor targeting is highly valuable because it will improve the tumor-absorbed dose and effectiveness of the treatment. New microcatheters used in interventional radiology allow for more selective angiography, delivering higher activities in the vicinity of tumors and sparing the healthy liver. Interventional radiologists are able to perform this kind of selective approach more and more, splitting the activity among multiple injections for the different arterial branches of the tumor [54]. For this purpose, a cone-beam CT can be performed during the liver arteriography for precisely identifying the feeding arteries of a tumor [55].

Moreover, the innovative new anti-reflux catheters could also improve tumor targeting. In a retrospective analysis of neuroendocrine and HCC tumors, the anti-reflux catheters were found to provide significantly better tumor targeting than the classic end-hole catheters [56]. Some drugs infused during the treatment can also increase the tumor-to-normal-liver ratio (i.e., the tumor targeting). The co-infusion of angiotensin II during SIRT was also shown to significantly increase tumor targeting (tumor-to-normal-liver ratio × 3) by decreasing the healthy liver arterial flow, while the tumor arterial flow increased [57]. However, this effect was short-lived (a few minutes) and rapidly reversed despite the continuous infusion of angiotensin II due to liver arterial vasodilation triggered by the low arterial flow (i.e., a vascular escape mechanism) [58].

For clinical application of SIRT, the arterial vasoconstriction needs to be longer to facilitate injection of all radioactive microspheres before activation of this opposing effect. Alternative drugs, such as sodium acetate and dopexamine, could induce a longer vasoconstrictive effect in the liver artery [59]. These mesenteric vasodilators induce an increase in portal blood flow, resulting in a reflex vasoconstriction of the liver artery (i.e., the hepatic arterial buffer response) [60], an effect to which tumors are not susceptible due to their anarchic vascularization. Hence, the arterial flow would be redirected in tumors preferentially, and the tumor-to-normal-liver ratio would be increased. For this purpose, dopexamine seems to be a good candidate. This analogue of dopamine is responsible for vasodilation of the mesenteric arteries, inducing a reduction in the liver arterial flow to a factor of four in an animal model [61]. Moreover, this drug has a short half-life and is well-tolerated at low infusion rates [62]. Future investigations are needed to evaluate this effect more thoroughly.

## 6. Good HCC Candidates for SIRT

The collective research efforts have provided a good understanding of the factors responsible for treatment ineffectiveness in HCC, helping clinicians to select the best candidates for SIRT. Currently, using tumor dosimetry, MAA imaging can generally select patients who will respond well to SIRT (high tumor uptake and high absorbed dose) or those who will not respond (low tumor uptake, low absorbed dose) [63]. The interest of this dosimetry applied to MAA SPECT/CT was confirmed in the recent DOSISPHERE randomized controlled trial [40] and was also well-illustrated in a retrospective study of 41 patients treated for advanced HCC with portal vein thrombosis. The overall survival was only 4.3 mo when the tumor-absorbed dose was less than 205 Gy and 18.2 mo when at least 205 Gy (glass microspheres) [49]. Moreover, patients with portal vein thrombosis and poor targeting via MAA imaging had a very poor prognosis.

HCC is a heterogeneous group of tumors with different behaviors; some can be very aggressive, with a tumor doubling time ranging from 3 mo to 1 year [64]. [^18^F]-Fluorodeoxyglucose (FDG) PET/CT has low sensibility, with a significant uptake in less than 50–65% of the cases [65]. However, data have indicated that HCC tumors with high [^18^F]FDG uptake are more aggressive, with patients at higher risk of recurrence and poorer survival [66]. SIRT is less effective in this population, with a significant reduction of the local control, progression-free survival (PFS) and overall survival (OS) [67,68]. In advanced HCC, randomized trials have failed to demonstrate a superior PFS and OS in patients treated by RE compared to sorafenib despite a significant increase of the tumor response rate in the RE arm (Table 1). Loco-regional therapies such as SIRT may be less effective for patients with aggressive HCC tumors, and [^18^F]FDG PET/CT could be useful to identify these patients. Decompensated liver function is also a strong predictor of poor survival. The baseline bilirubin level, the Child–Pugh score, and the albumin–bilirubin grade were independent predictors of poor survival in patients treated with SIRT [50,69,70]. The median overall survival rates reported for advanced HCC patients treated with sorafenib range from 6.5 mo to 14.7 mo [71,72,73,74]. To compare, some markers of poor prognosis have been identified in large retrospective studies of advanced HCC patients treated with SIRT (Table 6). Patients with poor performance status (ECOG 2 or more), extrahepatic metastases, portal vein thrombosis extending to the main left/right branch, tumor burden > 50% of the liver volume, and a baseline alteration of the liver function (albumin–bilirubin score of 3 or bilirubin level of 2–3 mg/dL) have reported median survival rates that fall between 4.3 and 8.2 mo (Table 6). Lescure et al. demonstrated also a strong correlation between the ALBI score (grade 2 or 3) and the risk of REILD [75].

In these groups of patients, RE would be ineffective and potentially toxic; alternative systemic therapies should be suggested.

## 7. Conclusions

SIRT is an effective therapy in HCC and can significantly improve the outcome of patients. Dosimetry plays a key role in predicting its effectiveness and can be optimized using a personalized method of activity planning (i.e., multi-compartmental dosimetry). Selection of patients based on performance status, liver function, tumor characteristics, and tumor targeting as assessed by MAA imaging can also improve the clinical performance of SIRT.

## Figures and Tables

**Table 1:** Prospective and randomized studies in hepatocellular carcinoma.

Studies	Groups	Nb of Patients	BCLC Score	Adverse Events (≥Grade 3)	RR	TTP (mo)	PFS (mo)	OS (mo)
Pitton et al., 2015 [22]	SIRT (resin)	12	B: 100%	NA	NA	12.4	6	19.7
	TACE	12	A: 8% B: 92%	NA	NA	11.2	7.2	26.3
Salem et al., 2016 [17]	SIRT (glass)	24	A: 75% B: 25%	NA	87%	>26 *	NA	18.6
	TACE	21	A: 81% B: 19%	NA	74%	4.8	NA	17.7
SARAH [19]	SIRT (resin)	237	C: 100%	41%	19% *	NA	4.1	9.9
	Sorafenib	222	C: 100%	63% *	12%	NA	3.7	9.9
SIRveNIB [20]	SIRT (resin)	130	B: 61% C: 39%	28%	23% *	6.1	6.3	8.8
	sorafenib	162	B: 54% C: 45%	51% *	2%	5.4	5.2	10
SORAMIC [23]	SIRT (resin) + sorafenib	114	A: 4% B: 28% C: 68%	65% *	NA	NA	NA	14
	sorafenib	174	A: 2% B: 28% C: 70%	54%	NA	NA	NA	11.1

* Statistically significant differences using a Kaplan–Meier method and the log-rank test (*p* < 0.05). Nb, Number; mo, months; BCLC, Barcelona Clinic Liver Cancer; NA, not available; OS, overall survival; PFS, progression-free survival; RR, response rate; SIRT, selective internal radiation therapy; TACE, transarterial chemoembolization; RR, response rate; TTP, time to progression.

**Table 2:** Main studies reporting a correlation between tumor dosimetry in SIRT and clinical response.

Study	Study Design	Type of Microspheres	Nb of Patients	Correlation with Radiological Response	Correlation with PFS	Correlation with OS
Strigari et al., 2010 [27]	Retrospective	Resin	73	✓	NA	NA
Chiesa et al., 2011 [31]	Retrospective	Glass	46	✓	NA	NA
Garin et al., 2012 [32]	Retrospective	Glass	36	✓	✓	✓
Garin et al., 2017 [33]	Retrospective	Glass	85	✓	NA	✓
Kappadath et al., 2018 [34]	Retrospective	Glass	34	✓	NA	NA
Allimant et al., 2018 [35]	Retrospective	Resin	38	✓	✓	NA
Chan et al., 2018 [36]	Prospective	Glass	27	✓	NA	NA
Hermann et al., 2020 [28]	Prospective ^+^	Resin	121	✓	NA	✓
Dewaraja et al., 2020 [29]	Retrospective	Glass	28	✓	NA	NA
d’Abadie et al., 2021 [37]	Retrospective	Resin and glass	45	✓	✓	✓
Son et al., 2021 [38]	Prospective ^+^	Resin	34	✓	NA	NA
Nodari et al., 2021 [39]	Retrospective	Resin and glass	48	✓	NA	✓
Garin et al., 2021 [40]	Prospective, randomized, multicenter	Glass	56	✓	✓	✓

Nb, Number; OS:, overall survival; PFS, progression-free survival; ✓, significant correlation with tumor dosimetry; NA, not available. ^+^ Secondary analysis of prospectively acquired data.

**Table 3:** Main studies reporting threshold absorbed doses correlated with clinical outcome in hepatocellular carcinoma using glass microspheres.

Study	Nb of Patients	Nb of Tumors	Dosimetry Performed with	Criteria for Radiological Response Assessment	TD Threshold for Radiological Response	Median PFS above and under the TD Threshold	Median OS above and under the TD Threshold
Chiesa et al., 2011 [31]	46	91	MAA SPECT/CT	EASL	257 Gy (Se: 85%, Sp: 70%)	NA	NA
Garin et al., 2012 [32]	36	58	MAA SPECT/CT	EASL	205 Gy (Se: 100%, Sp: 75%)	14 mo vs. 5.2 mo *	18 mo vs. 9 mo *
Garin et al., 2017 [33]	85	132	MAA SPECT/CT	EASL	205 Gy (Se: 98%, Sp NA)	NA	21 mo vs. 6.5 mo *
Kappadath et al., 2018 [34]	34	53	^90^Y SPECT/CT	modified RECIST 1.1	160 Gy (50% response)	NA	NA
Chan et al., 2018 [36]	27	38	^90^Y PET/CT	modified RECIST 1.1	200 Gy (Se: 66%, Sp: 100%)	NA	NA
d’Abadie et al., 2021 [37]	26	73	^90^Y PET/CT	modified RECIST 1.1	118 Gy (Se: 93%, Sp: 75%)	5.5 mo vs. 1.8 mo *	14.6 mo vs. 5.5 mo *
Nodari et al., 2021 [39]	23	NA	^90^Y PET/CT	NA	156 Gy (Se and Sp NA)	NA	23 mo vs. 14 mo *

* Statistically significant differences using a Kaplan–Meier method and the log-rank test (*p*-value < 0.05). NB, Number; mo, months EASL, European Association for the Study of the Liver; MAA SPECT/CT, technetium-99m macro-aggregated albumin single-photon emission computed tomography combined with computed tomography; NA, not available; OS:, overall survival; PFS, progression-free survival; RECIST, Response Evaluation Criteria in Solid Tumors; Se, sensitivity; SIRT, selective internal radiation therapy; Sp, specificity; TD, tumor-absorbed dose threshold; ^90^Y PET/CT, yttrium-90 positron emission tomography combined with computed tomography; ^90^Y SPECT/CT, yttrium-90 single-photon emission computed tomography combined with computed tomography.

**Table 4:** Main studies reporting threshold absorbed doses correlated with clinical outcome in hepatocellular carcinoma using resin microspheres.

Study	Nb of Patients	Nb of Tumors	Dosimetry Performed with	Criteria for Radiological Response Assessment	TD Thresholdfor Radiological Response	Median PFS above and under the TD Threshold	Median OS above and under the TD Threshold
Allimant et al., 2018 [35]	38	42	^90^Y PET/CT	modified RECIST 1.1	61 Gy (Se: 76%, Sp: 75%)	12.1 mo vs. 6.3 mo *^+^	NA
Hermann et al., 2020 [28]	121	NA	MAA SPECT/CT	RECIST 1.1	100 Gy (72% response)	NA	14.1 mo vs. 6.1 mo *
d’Abadie et al., 2021 [37]	19	60	^90^Y PET/CT	modified RECIST 1.1	61 Gy (Se: 87%, Sp: 64%)	4.6 mo vs. 1.6 mo *	16 mo vs. 5.3 mo *
Son et al.,2021 [38]	34	45	MAA SPECT/CT	modified RECIST 1.1	125 Gy (Se: 86%, Sp: 75%)	NA	NA
Nodari et al., 2021 [39]	25	NA	^90^Y PET/CT	NA	98 Gy (Se and Sp NA)	NA	23 mo vs. 14 mo *

* Statistically significant differences using a Kaplan–Meier method and the log-rank test (*p*-value < 0.05). ^+^ Reported for complete tumor targeting (25 patients). Nb, Number; mo, months; EASL, European Association for the Study of the Liver; MAA SPECT/CT, technetium-99m macro-aggregated albumin single-photon emission computed tomography combined with computed tomography; NA, not available; OS, overall survival; PFS, progression-free survival; RECIST, Response Evaluation Criteria in Solid Tumors; Se, sensitivity; SIRT, selective internal radiation therapy; Sp, specificity; TD, tumor-absorbed doses; ^90^Y PET/CT, yttrium-90 positron emission tomography combined with computed tomography; ^90^Y SPECT/CT, yttrium-90 single-photon emission computed tomography combined with computed tomography.

**Table 5:** Main results of the DOSISPHERE-01 randomized controlled trial [40].

	Personalized Dosimetry	Standard Dosimetry
Number of patients	28	28
Activity planned in GBq, median	3.6 *	2.6
Response rate at 3 mo, EASL criteria	71% *	36%
Curative surgery intent after SIRT	36% *	4%
REILD	9%	10%
Overall survival in mo, median	26.6 ^+^	10.7

* Statistically significant differences using a chi-square or Fisher’s exact tests (*p* < 0.05). ^+^ Statistically significant differences using a using a Kaplan–Meier method and the log-rank test (*p* < 0.05). EASL, European Association for the Study of the Liver; SIRT, selective internal radiation therapy; REILD, radioembolization-induced liver disease.

**Table 6:** Studies reporting factors of poor prognosis in advanced HCC treated by SIRT.

Study	Nb of Patients	Parameter Related to Worse Prognosis	Median Survival (95% CI Interval)
Ali et al., 2018 [76]	547	ECOG 2	4.3 mo (2.5–7.8)
		Extrahepatic metastases	7.4 mo (6.0–9.0)
		PVT	7.3 mo (6.3–8.0)
Spreafico et al., 2018 [50]	120	Bilirubin > 1.2 mg/dL	9.5 mo (8.8–10.2)
		PVT extended to right/left main branch	8.2 mo (5.7–10.8)
		Tumor burden > 50% liver volume	6.4 mo (5.2–7.6)
Abouchaleh et al., 2018 [46]	185	ECOG 2	2.5 mo (2–4.6)
		Bilirubin 2–3 mg/dL	5 mo (2.2–9.7)
		PVT extended to right/left main branch	7.7 mo (5.3–10.4)
Antkowiak et al., 2019 [69]	541	Bilirubin 2–3 mg/dL	8 mo (6.7–21)
		ALBI grade 3	6.7 mo (5.7–8.8)
Zu et al., 2020 [47]	91	CHILD B7	6 mo (4.4–7.6)
Lescure et al., 2021 [75]	222	ALBI grade 3	8.1 mo (4.1–12.1)

Nb, number; mo, months; ALBI, albumin–bilirubin; ECOG, Eastern Cooperative Oncology Group; PVT, portal vein thrombosis.
